# Exploring Factors Associated With Missed Dental Appointments: A Machine Learning Analysis of Electronic Dental Records

**DOI:** 10.7759/cureus.47304

**Published:** 2023-10-19

**Authors:** Hussam M Alqahtani, Yasmine N Alawaji

**Affiliations:** 1 Preventive Dental Science, College of Dentistry, King Saud Bin Abdulaziz University for Health Sciences, Riyadh, SAU; 2 Research and Development, King Abdullah International Medical Research Center, Riyadh, SAU; 3 Dental Hospital, Ministry of National Guard Health Affairs, Riyadh, SAU

**Keywords:** machine learning, dental school, electronic health records, appointment adherence, dental appointment

## Abstract

Background: This study aimed to employ machine learning techniques to explore the factors that could be associated with missed dental appointments.

Methods: This cross-sectional study analyzed a total of 14,066 electronic dental records. Dental appointment adherence was categorized as attended or missed. Descriptive statistics and machine learning techniques, including conditional inference regression trees (CTree) and random forests (RFs), were employed for the analyses.

Results: About 31% of dental appointments were missed. Among the study population, appointments scheduled on Monday of the first week in the school year had the highest percentage of missed appointments, reaching up to 60%. Similarly, appointments scheduled on weeks 9, 10, 15-19, on Mondays, and with female dental students had slightly above 40% of missed appointments. The random forest analysis identified the week of the dental appointment, age, clinical day, and dental education level of students as the most influential variables in predicting dental appointment adherence.

Conclusions: The most significant factors associated with a higher proportion of missed dental appointments were scheduled during specific weeks, on Mondays, with younger patients (<50 years), and with female dental students. Therefore, identifying these factors may assist healthcare providers and dental institutions in planning strategies to improve appointment attendance.

## Introduction

Missed dental appointments may have a substantial impact on patients and the dental care system. Additionally, they may hinder the clinical training of dental students. Studies have shown a remarkable occurrence of missed dental appointments [1-3]. However, the use of a self-administered questionnaire as a primary source for data collection could be biased due to potential recall inaccuracies or participation bias. A more dependable data source was utilized in a study by performing a descriptive analysis of electronic dental records from 15,193 patients, and it found that 9% of the patients missed their appointments, 71% attended them, and 20% cancelled them [1].

The factors that could explain dental appointment adherence were not thoroughly examined in the literature. The use of descriptive analyses in previous studies allowed for an understanding of the pattern of dental appointment adherence. However, it had limited potential for exploring the factors associated with missed dental appointments without prior assumptions. Alternatively, the use of advanced analytical approaches in machine learning techniques, such as conditional inference regression tree (CTree) and random forest (RF), can be a more compelling approach to exploring the factors associated with adherence to dental appointments.

Machine learning analyses have proven useful in identifying complex patterns in health records, as demonstrated in previous research on periodontal disease [4,5]. In one study, machine learning analysis allowed for the finding that periodontal disease was associated with sociodemographic factors and functional limitations rather than chronic conditions or geriatric syndromes [5]. Additionally, previous research has demonstrated that machine learning analysis can reveal new combinations of factors associated with a particular health issue without prior hypotheses [6,7]. Due to the limited background literature on factors that could impact adherence to dental appointments, the aim of this study was to employ machine learning techniques such as CTree and RF to explore the factors associated with missed dental appointments.

## Materials and methods

Data source

This cross-sectional study utilized the SALUD (Two-Ten Health, Ireland) electronic dental records system for 19 weeks between August 2022 and January 2023 at the College of Dentistry, King Saud bin Abdulaziz University for Health Sciences. Patients who were ≥20 years old and had scheduled dental appointments with senior undergraduate dental students were included. The study excluded cancelled appointments by dental students, walk-ins, emergency visits, or appointments scheduled during final exam weeks (12th, 13th, and 14th). Ethics approval was obtained from the institutional review board of the King Abdullah International Medical Research Center (#NRC23R/350/06).

Data collection

We collected demographic variables including age, grouped into a total of 11 5-year cohorts, gender (male or female), and the level of dental student training (third or fourth year) based on a four-year dental program. Additionally, we categorized the patient's location in relation to their residence in Riyadh city according to the regions of Riyadh province, which encompassed the north, west, center, south, and east. Regions outside of Riyadh or those with inaccurate labeling were classified as 'other'. Furthermore, we recorded additional appointment details such as 1st-19th weeks, excluding the 12th, 13th, and 14th weeks, appointment time (morning or afternoon), and day of the week (Sunday to Thursday). The primary outcome of interest in our study was dental appointment adherence, which was classified as either attended or missed.

Statistical analyses

A descriptive analysis was conducted, presenting the patterns of dental appointments. To address the research question, we utilized two machine learning methods: CTree and random forest. The adopted procedures were consistent with previous studies on CTree and RF [4,5,7]. In CTree, a maximum tree depth of five splits, a minimum terminal node size of 100 participants, and a p-value threshold of 0.001 were utilized as stopping criteria. For the random forest, we generated 3,000 trees and randomly selected three explanatory variables at each node split for each model. The analysis was performed using R version 3.6 and the 'partykit' (CTree) and 'randomForest' (random forest) packages (RStudio Team, USA).

## Results

The characteristics of dental appointments stratified by attendance and missed appointment status, as well as the distribution of dental appointment adherence across weekdays and week numbers, were illustrated in Tables 1-2, respectively. Out of the 14,066 dental appointments, 31.06% were missed. Among the study population, individuals aged 25-34 years and those with appointments scheduled on Mondays and Tuesdays had a higher likelihood of missing their appointments. Patients treated by third-year female dental students, those living in the north, center, and west regions of Riyadh province, and those seen during the 1st, 8th-10th, and 15th-19th weeks were also more likely to miss their appointments. On the other hand, elderly individuals aged 50 years and older exhibited a higher attendance rate compared to younger people, with specific age groups such as 50-54 years and 60-64 years having the lowest proportion of missed appointments. Moreover, appointments scheduled with third-year male dental students, individuals residing in the south of Riyadh province, and during weeks 4 and 11 had significantly lower rates of missed appointments.

**Table 1:** Characteristics of the study population

Variables	Appointment adherence		
	Attended	Missed	Total
No. of dental appointments	9697	4369	14,066
Age, N (%)			
<20	2666 (68.55)	1223 (31.45)	3889
20–24	1590 (67.12)	779 (32.88)	2369
25–29	854 (65.90)	442 (34.10)	1296
30–34	898 (66.37)	455 (33.63)	1353
35–39	851 (70.98)	348 (29.02)	1199
40–44	953 (70.49)	399 (29.51)	1352
45–49	614 (68.76)	279 (31.24)	893
50–54	563 (75.27)	185 (24.73)	748
55–60	274 (70.80)	113 (29.20)	387
60–64	246 (77.36)	72 (22.64)	318
>65	188 (71.76)	74 (28.24)	262
Sex, N (%)			
Male	5241 (69.68)	2280 (30.32)	7521
Female	4456 (68.08)	2089 (31.92)	6545
Nationality, N (%)			
Saudi	8253 (68.84)	3736 (31.16)	11989
Non-Saudi	1444 (69.52)	633 (30.48)	2077
Level of dental students N (%)			
FD3	2133 (65.67)	1115 (34.33)	3248
FD4	2895 (68.26)	1346 (31.74)	4241
MD3	1866 (73.52)	672 (26.48)	2538
MD4	2803 (69.40)	1236 (30.60)	4039
Patient’s location in relation to residence in Riyadh city, N (%)			
North	825 (66.00)	425 (34.00)	1250
West	328 (63.69)	187 (36.31)	515
Center	143 (64.71)	78 (35.29)	221
South	447 (74.62)	152 (25.38)	599
East	4179 (69.25)	1856 (30.75)	6035
Other	3775 (69.32)	1671 (30.68)	5446
Time of the clinical session, N (%)			
AM	5343 (68.02)	2512 (31.98)	7855
PM	4354 (70.10)	1857 (29.90)	6211

**Table 2:** Distribution of dental appointment adherence across weekdays and week number *During the 11th week, only senior students were allowed to schedule patients. During the 12th-14th weeks, dental students had final exams.

Variables	Appointment adherence	Attended	Missed	Total
No. of dental appointments		9697	4369	14,066
Day of the week, N (%)	Sunday	1857 (70.66)	771 (29.34)	2628
	Monday	2149 (66.66)	1075 (33.34)	3224
	Tuesday	1456 (65.94)	752 (34.06)	2208
	Wednesday	1998 (70.03)	855 (29.97)	2853
	Thursday	2237 (70.95)	916 (29.05)	3153
Week number, N (%)	1	402 (59.82)	270 (40.18)	672
	2	649 (72.92)	241 (27.08)	890
	3	696 (72.27)	267 (27.73)	963
	4	423 (73.69)	151 (26.31)	574
	5	746 (72.92)	277 (27.08)	1023
	6	726 (71.18)	294 (28.82)	1020
	7	736 (72.94)	273 (27.06)	1009
	8	415 (66.29)	211 (33.71)	626
	9	751 (68.03)	353 (31.97)	1104
	10	792 (66.67)	396 (33.33)	1188
	11^*^	317 (81.07)	74 (18.93)	391
	15	612 (69.00)	275 (31.00)	887
	16	722 (63.28)	419 (36.72)	1141
	17	580 (64.02)	326 (35.98)	906
	18	757 (66.93)	374 (33.07)	1131
	19	373 (68.95)	168 (31.05)	541

**Figure 1:**
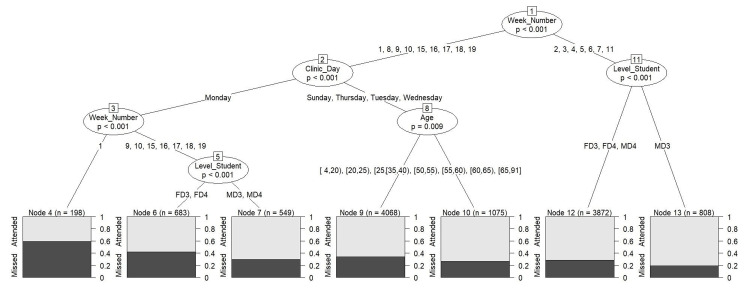
The conditional inference regression tree analysis to identify factors associated with the proportion of missed dental appointments.

Based on the CTree analysis, certain factors were associated with the proportion of missed dental appointments, such as appointments scheduled on Monday of the first week of the school year, which were found to have the highest percentage of missed appointments, reaching up to 60% (nodes 1, 2, 3, and 4), as shown in Figure 1. Furthermore, appointments scheduled on weeks 9, 10, 15-19, on Mondays, and with female dental students had slightly above 40% of missed appointments, as per nodes 1, 2, 3, and 5. In contrast, appointments scheduled in specific weeks (2nd-7th, and 11th) with the third-year male dental students had the lowest percentage of missed appointments at 20% (nodes 1, 11, and 13). In addition, a low proportion of missed appointments, at 25%, was observed for appointments scheduled during weeks 1st, 2nd-10th, and 15th-19th, on days other than Monday, and among individuals ≥50 years.

**Figure 2:**
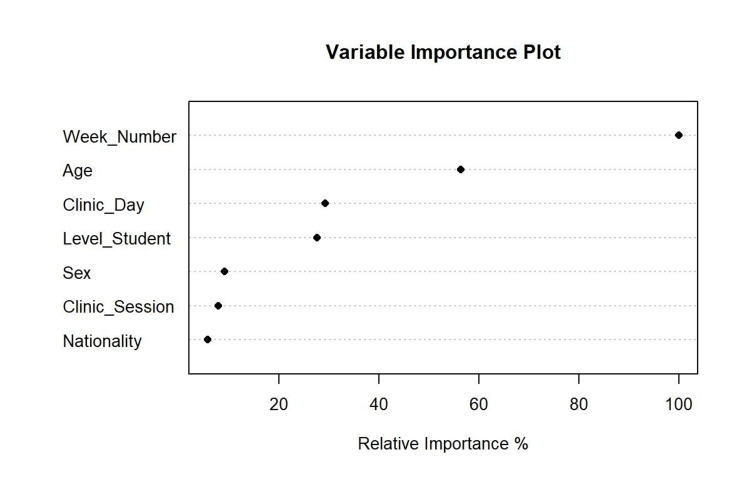
Random forest plot ranking the most influential factors for attended and missed dental appointments.

The ranking of the most influential variables by random forest for attended and missed dental appointments is displayed in Figure 2. The analysis identified the week of the dental appointment, age, clinical day, and level of dental education as the most important variables. These variables also feature in the CTree model, affirming their significant impact on dental appointment status.

## Discussion

This study utilized innovative machine learning methods (CTree and random forest) to investigate the factors influencing attended and missed dental appointments. The analysis revealed that the week of the dental appointment, the day of the clinical session, and the dental education level of students were the key factors associated with attendance and missed appointment status. Building upon prior research by Alqahtani and Alawaji [1], who used electronic dental records to describe appointment patterns, our study employed these methods to automatically select variables, detect interactions, and uncover non-linear relationships. CTree effectively captured complex relationships and identified specific combinations of factors strongly associated with missed appointments without preconceived assumptions [6,7]. Conversely, random forest employed a bootstrap aggregation approach to rank the importance of multiple variables simultaneously [6,7], allowing us to assess their relative significance.

We observed variations in the number of scheduled appointments across different weeks. The 5th-7th, 9th, 10th, 16th, and 18th weeks had the highest appointment numbers, while the 4th, 11th, and 19th weeks had the lowest. To accurately estimate missed dental appointments, our analysis considered specific days and weeks, taking into account factors such as long weekends and non-clinical days. The study covered a total of 19 weeks, following the three-semester system of the College of Dentistry at King Saud bin Abdulaziz University for Health Sciences. The first semester consisted of 10 weeks of clinical training, with an additional week (the 11th week) dedicated to senior students. The second semester comprised five weeks. The 12th-14th were excluded from the analysis as no clinical appointments took place during that period due to final exams and vacations. It is worth noting that certain weeks were affected by specific circumstances. For instance, in week one, there was no clinic on Sunday due to student orientation. The fourth week had no clinic on Wednesday and Thursday, resulting in an extended weekend. In the eighth week, the clinic was closed on Sunday and Monday due to a long weekend. Week 17 had no clinic on Sunday due to another long weekend, and in the 19th week, there was no clinic on Thursday due to unstable weather conditions.

About 31% of dental appointments were missed in the study. The use of electronic dental records and a larger sample size in this study provide a more reliable estimate of the percentage of missed appointments compared to previous studies. For example, one study reported a rate of 24% for missed appointments [2], while another study reported a higher rate of 58% [3]. In addition, the highest percentage of missed appointments, reaching 60%, occurred among those with appointments scheduled during the first week and on Mondays. The lack of a clinic on Sunday due to student orientation may have contributed to missed appointments, as students were unable to remind their patients of their upcoming appointments. Existing research consistently supports the significant role of personal reminders in positively influencing appointment attendance rates [1,8,9].

Interestingly, Monday, despite having the highest number of scheduled appointments compared to other days, had over one thousand missed appointments, accounting for about one-third of the total. This finding contradicts our previous study [1], which did not find any variations in appointment adherence based on clinical session time, day of the week, or gender. The use of machine learning analyses in the current study may have revealed previously unidentified patterns. Furthermore, appointments scheduled on the 9th, 10th, 15th-19th weeks, on Mondays, and with female dental students had a slightly above 40% of missed appointments. It is worth noting that one possible reason for this could be that one-third of dental appointments with female students (n=2461) were missed, compared to appointments with male students (n=1908), even though the number of scheduled appointments with female students (n=7489) was higher than that with male students (n=6577).

Regarding attended dental appointments, appointments scheduled in specific weeks (2nd-7th and 11th) with third-year male dental students had the highest attendance percentage, reaching 80%. Additionally, it is noteworthy that the attendance rate for each of these weeks individually was at least 71%. Furthermore, CTree analyses revealed that older individuals (≥50 years) displayed a higher attendance rate of approximately 75% compared to younger people. This difference can be partially explained, as supported by our previous research [1], by the lower proportion of older individuals in comparison to younger individuals.

Surprisingly, the patient's location in relation to the residence in Riyadh city did not emerge as a significant factor in both machine learning models, indicating that it was not associated with attended and missed dental appointments. However, it is worth noting that there was a considerable discrepancy in the proportion of people residing in different regions of Riyadh. For instance, the number of people living in the south, west, and central regions of Riyadh ranged from 200 to 600, while the north region had 1250 people, and there were over 5400 people residing in the east and other regions.

This study has several significant aspects. To the best of our knowledge, this is the first study to utilize machine learning analyses for analyzing electronic dental records in Saudi Arabia. The study involved a sample size of 14,066 dental appointments, which provided the opportunity to employ innovative automated machine learning analyses to discover valuable insights. The implication of the present study is that it could potentially serve as a benchmark for future research, given the statistical methods employed and the data sources utilized.

Among the limitations of this study, variables with unequal numbers of appointments were included, which may impact the analyses and interpretation of the results. The study was restricted by the available data in the electronic dental records, which may have limited the depth of our analysis. In addition, the study was conducted at a single center, which may restrict the generalizability of the findings. Further studies that consider additional variables, such as whether appointments were scheduled with the same operator, could provide further valuable insights. In addition, further investigation of the factors we found in this study may help in planning strategies that could improve dental appointment adherence.

## Conclusions

Our study identified several factors associated with missed dental appointments. Appointments scheduled during specific weeks, on Mondays, with younger individuals (<50 years), and with female dental students were found to have a higher percentage of missed appointments. Further investigation of these factors may help in designing strategies to improve appointment attendance rates and optimize dental care delivery.
